# Analysis of Amyloid Precursor Protein Function in *Drosophila melanogaster*

**DOI:** 10.3389/fnmol.2016.00061

**Published:** 2016-07-26

**Authors:** Marlène Cassar, Doris Kretzschmar

**Affiliations:** Oregon Institute of Occupational Health Sciences, Oregon Health and Science UniversityPortland, OR, USA

**Keywords:** *Drosophila melanogaster*, amyloid precursor proteins, neuronal outgrowth, neuronal survival, synaptogenesis

## Abstract

The Amyloid precursor protein (APP) has mainly been investigated in connection with its role in Alzheimer’s Disease (AD) due to its cleavage resulting in the production of the Aβ peptides that accumulate in the plaques characteristic for this disease. However, APP is an evolutionary conserved protein that is not only found in humans but also in many other species, including *Drosophila*, suggesting an important physiological function. Besides Aβ, several other fragments are produced by the cleavage of APP; large secreted fragments derived from the N-terminus and a small intracellular C-terminal fragment. Although these fragments have received much less attention than Aβ, a picture about their function is finally emerging. In contrast to mammals, which express three APP family members, *Drosophila* expresses only one APP protein called APP-like or APPL. Therefore APPL functions can be studied in flies without the complication that other APP family members may have redundant functions. Flies lacking APPL are viable but show defects in neuronal outgrowth in the central and peripheral nervous system (PNS) in addition to synaptic changes. Furthermore, APPL has been connected with axonal transport functions. In the adult nervous system, APPL, and more specifically its secreted fragments, can protect neurons from degeneration. APPL cleavage also prevents glial death. Lastly, APPL was found to be involved in behavioral deficits and in regulating sleep/activity patterns. This review, will describe the role of APPL in neuronal development and maintenance and briefly touch on its emerging function in circadian rhythms while an accompanying review will focus on its role in learning and memory formation.

The Amyloid precursor protein (APP) is a key factor in Alzheimer’s Disease (AD) because, as the name implies, it is the precursor from which the neurotoxic Aβ peptides are generated (Glenner and Wong, 1984; Masters et al., 1985). APP is a type-one membrane-spanning protein consisting of a large extracellular N-terminal domain and a small intracellular C-terminal domain in addition to the Aβ region (Goldgaber et al., 1987; Kang et al., 1987; Robakis et al., 1987; Tanzi et al., 1987). Alternative splicing of the APP gene produces three major isoforms (695aa, 751aa, and 770aa), with APP_695_ being the major form found in the nervous system (Tanaka et al., 1989; Lorent et al., 1995). In addition to APP, vertebrates express two closely related proteins called Amyloid Precursor-Like Proteins (APLP) 1 and 2 (Coulson et al., 2000; Turner et al., 2003).

Over the last decade, transgenic *Drosophila* expressing either human APP_695_ or Aβ have been extensively used to study the pathogenic function of APP (Cowan et al., 2010; (Iijima-Ando and Iijima, 2010; Moloney et al., 2010; Wentzell and Kretzschmar, 2010; Prüßing et al., 2013; Bouleau and Tricoire, 2015). However, insects also express an ortholog of APP which was named APP-like or APPL. APPL is about 30% overall identical to human APP_695_ but a much higher degree of conservation is found in the extracellular E1 and E2 domains and especially in the C-terminal intracellular domain or AICD (Rosen et al., 1989; Swanson et al., 2005; Figure 1). Five isoforms of APPL are described in *Drosophila* that range from 830aa to 890aa (Attrill et al., 2016), however it is unknown whether these isoforms are functionally different. In contrast to the human protein, which is also expressed in non-neuronal cells (Sandbrink et al., 1994a,b), APPL is only expressed in neurons, starting at stage 13 of *Drosophila* embryogenesis (Luo et al., 1990; Martin-Morris and White, 1990). Interestingly, APPL lacks the Kunitz-like domain and is therefore more closely related to APP_695_ than other isoforms (Arai et al., 1991). Like APP, APPL is processed by several secretases, resulting in secreted fragments, a neurotoxic Aβ-like peptide, and an intracellular AICD (Luo et al., 1990; Carmine-Simmen et al., 2009; Bolkan et al., 2012). However, in comparison to APP, the cleavage sites for the α- and β-secretase are reversed in APPL, with the β-site being more proximal to the transmembrane region and the α-site being more distal (Carmine-Simmen et al., 2009; Stempfle et al., 2010). The evolutionary conservation of APPL and its processing not only suggests that this protein has important physiological functions but also that studies in *Drosophila* can provide insights into the normal functions of human APP and its proteolytic fragments.

**Figure 1:**
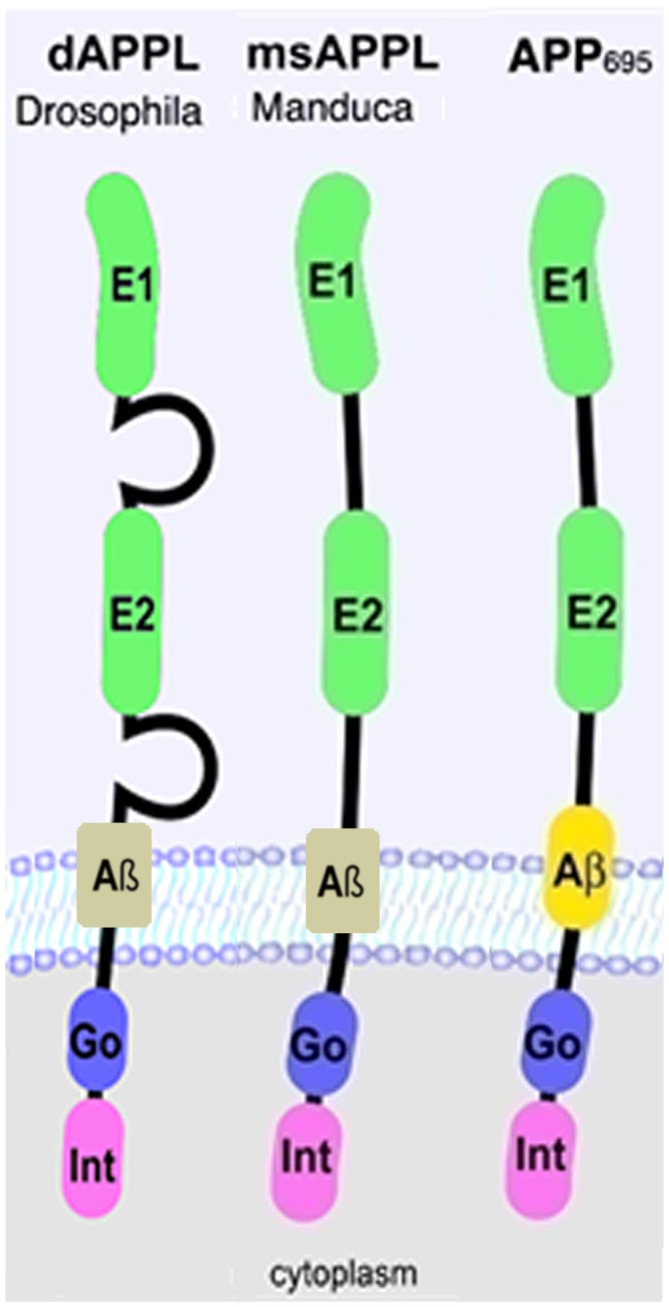
**Schematic representation of human APP_695_ and insect Amyloid Precursor Protein-like (APPL).** Go, Goα-binding site; Int, internalization domain; E, extracellular domain 1 and 2.

## APPL and the Development of the Peripheral Nervous System

Flies that completely lack APPL (*Appl*^d^, Luo et al., 1992) are viable but show a loss of sensory bristles on the sternopleuron and scutellum, parts of the adult thorax (Merdes et al., 2004). The same phenotype was observed when knocking down the *Appl* mRNA during development via RNA-interference. These mechano-sensory organs (MSOs) are derived from a sensory organ precursor cell (SOP), which is determined by lateral inhibition via Notch signaling. They consist of a shaft, a socket, a sheath cell, the sensory neuron, and a supporting glial cell (Lai and Orgogozo, 2004). Because not only the sensory neuron is missing in *Appl^d^* flies, but also the external cell types of the MSO, this indicates that APPL plays a role in SOP linage formation (Merdes et al., 2004). This result implies that in the peripheral nervous system (PNS) APPL is expressed in neuronal precursor cells, possibly playing a role in the determination of the MSOs, whereas in the central nervous system (CNS) it is restricted to differentiated neurons (Luo et al., 1990).

In addition, APPL is required for the correct development of the enteric nervous system (ENS) in insects, more specifically in the migration of enteric neurons. During embryonic development of *Manduca sexta*, the neurons in the enteric plexus (EP cells) align with the muscle bands on the midgut and foregut and subsequently migrate along these pathways (Copenhaver and Taghert, 1989). APPL expression is detectable in the EP cells starting shortly before the onset of migration (Swanson et al., 2005) and knocking down APPL caused the EP neurons to ectopically migrate onto the interband regions (Ramaker et al., 2013). In *Drosophila*, the enteric neurons do not migrate along the gut and therefore this function of APPL does not play a role in flies. If APPL can act as a neuronal guidance receptor in cell migration of other neurons in flies remains to be determined.

## APPL and Neuronal Outgrowth

The expression of APPL during embryonic development correlates with the onset of axonal outgrowth and it is especially abundant in growing axons and in areas of synapse formation (Luo et al., 1990; Martin-Morris and White, 1990). Initially, no gross abnormalities were described in the larval or adult CNS of *Appl^d^* flies. However, they showed behavioral deficits in the fast-phototaxis assay, which is based on visual input and startle-induced locomotion (Luo et al., 1992). Later studies revealed that the loss of APPL does have effects on neuronal outgrowth, although the phenotypes are more subtle. Using cultures derived from embryonic neuroblasts, Li et al. (2004) found that the loss of APPL did not affect the initial outgrowth but resulted in significantly shorter neurites when cultured for 6 days. Surprisingly, overexpression of APPL or a secreted N-terminal fragment reduced neurite length whereas expression of a secretion-deficient form (APPL^sd^) or a variant that in addition lacks the intracellular C-terminus (APPL^delCT^) increased neurite length. Thus, in cell culture secreted APPL seems to function as a growth limiting ligand for a yet unknown receptor, whereas full-length APPL may act as a receptor that promotes neurite growth. Focusing on specific cell types, changes in axonal outgrowth and arborization were also observed *in vivo*. Induction of APPL in the lateral neurons, a group of neurons that play a key role in the regulation of circadian rhythms, promoted axonal arborization, as did expression of human APP (Leyssen et al., 2005). Interestingly, in these experiments the C-terminus appeared to be required for the axonal outgrowth. Deleting the C-terminus of APP or only the YENPTY motif, which mediates the interaction with various proteins like X11α or Fe65 (Turner et al., 2003; Poeck et al., 2012), prevented these phenotypes.

Similarly, affecting the levels of APPL in the mushroom bodies caused changes in its morphology. The mushroom bodies are considered to be the center for learning and memory in flies. They consist of the calyx, which contains the dendrites and is localized in the dorsal-posterior part of the brain, and the peduncle, which is formed by the axons which project as a bundle from dorsocaudal to rostroventral (Heisenberg, 2003). These axons then separate and form five lobes with the α/α′ lobes projecting dorsally whereas the β/β′ and γ-lobes are horizontally orientated towards the midline of the brain. APPL is prominently expressed in the mushroom bodies, especially in the neurons that form the α and β lobes (Soldano et al., 2013). A function of APPL in these neurons was first suggested by Li et al. (2004) who showed that expressing additional APPL in the mushroom bodies resulted in a fuzzy appearance of the β-lobes, though only detectable in some flies. The authors suggested that this could be probably due to a loosened fasciculation of these axons. A more prominent phenotype was observed more recently by Soldano et al. (2013) analyzing *Appl^d^* flies. Although still not fully penetrant, 14% of these flies showed a complete loss of an α-lobe and 12% a loss of a β-lobe (Soldano et al., 2013). Interestingly, it turned out that APPL function is cell-autonomously required for the development of the β-lobe whereas its function in the α-lobe is non-autonomous. Rescue experiments showed that the C-terminus was required for the axonal outgrowth of the β-lobe (Soldano et al., 2013), as was suggested for the axonal growth of lateral neurons (Leyssen et al., 2005). In both cell types the function was mediated by the Abelson kinase, which binds to the C-terminus of APPL via the adapter protein disabled (Leyssen et al., 2005; Soldano et al., 2013). Result from the studies in mushroom body neurons suggested that this then regulates the activity of the Planar Cell Polarity signaling pathway (Soldano et al., 2013), a pathway that has been shown to regulate neuronal outgrowth in flies and vertebrates (Lyuksyutova et al., 2003; Ng, 2012). Notably, whereas these *in vivo* experiments show a requirement of the C-terminus, suggesting that APPL acts as a receptor in axonal outgrowth, the cell culture experiments indicated that the C-terminus is not needed to promote outgrowth. This might be due to the special conditions in culture or alternatively different neuronal subtypes use different fragments and signaling pathways for proper outgrowth.

It has also been shown that the loss of APPL affects the outgrowth of photoreceptors. APPL is expressed in all photoreceptors but a more prominent expression can be detected in the R7 and R8 subtype, whereby the expression depends on Ras signaling (Mora et al., 2013). R7 and R8 project into the medulla, the second optic neuropil in *Drosophila*, where they target different layers (Meinertzhagen and Hanson, 1993). Focusing on R7, Mora et al. (2013) found that 2% of the R7 cells do not reach their target field. Although this is a relatively mild phenotype, it nevertheless has physiological consequences because *Appl^d^* flies exhibited a reduced preference for UV light, which is detected by this photoreceptor subtype. Using a knock-down strategy for APPL, another group observed changes in the symmetrical arrangement of the photoreceptors in the adult eye combined with an occasional loss of R7 photoreceptors (Singh and Mlodzik, 2012). The authors also show that these phenotypes were enhanced by a knock down of *hibris (hbs)*, which is a family member of the immunoglobulin cell adhesion proteins (Johnson et al., 2012). HBS seems to exert its function by affecting the γ-processing of APPL because it can promote the cleavage of Presenilin (PSN) into its active form (Singh and Mlodzik, 2012). As in vertebrates, the fly γ-secretase consists of NCT, APH1, PEN2, and the catalytically active PSN (Hu and Fortini, 2003; Stempfle et al., 2010) and expression of *Drosophila* PSN was shown to promote APPL cleavage (Carmine-Simmen et al., 2009). The interaction of HBS with APPL therefore suggests that its function in photoreceptor development and outgrowth requires the C-terminus or more specifically C-terminal cleavage of APPL.

Together, these experiments show that APPL does have a function in neuronal development and outgrowth, most likely acting as a receptor for a so far unknown ligand. However, its loss neither prevents axonal growth nor are the phenotypes fully penetrant. This indicates that APPL acts more like a “robustness” factor that supports the correct outgrowth instead of initiating or allowing it.

## APPL Function in Synaptogenesis and Axonal Transport

In addition to affecting axonal growth, APPL has also been shown to interfere with synapse formation. During larval development, different types of synaptic boutons are added along the axonal terminus, forming the stereotyped pattern of neuromuscular junctions (NMJ) at the body wall muscles (Gramates and Budnik, 1999). *Appl^d^* mutant larvae revealed a significant reduction in bouton numbers whereas overexpression of APPL induced additional boutons of different sizes; large “parent” boutons and small “satellite” boutons that are connected to the parent boutons (Torroja et al., 1999). The C-terminus was required to induce this phenotype and interestingly a deletion of the YENPTY domain prevented the formation of satellite boutons. In contrast, a deletion of the G_0_ binding site (Figure 1) prevented the induction of additional parent boutons. These experiment suggest that APPL also acts as a receptor at the NMJ. Additional experiments showed that to fulfil its function at the NMJ, APPL interacts with the cell adhesion molecule Fasciclin II (Fas II; an neural cell adhesion molecule (NCAM) homolog; García-Alonso et al., 1995) and the PDZ-domain containing dX11/Mint protein (Hase et al., 2002; Ashley et al., 2005). Because dX11/Mint binds to the YENPTY domain, this would explain the requirement of the C-terminus of APPL for bouton formation (Ashley et al., 2005). dX11/Mint binding seems to regulate the localization of APPL because a loss of dX11/Mint or expression of a dX11/Mint construct with a deletion in the APPL binding site resulted in an increase in the levels of APPL at the boutons (Ashley et al., 2005). A role of dX11/Mint in regulating APPL localization was confirmed in mushroom body neurons where the loss of dX11/Mint caused a depletion of APPL from the axons in the peduncle and the lobes while mis-localizing it to the calyx, which contains the dendrites from which it is normally excluded (Gross et al., 2013).

As with photoreceptors, the defects in the formation of the NMJ may not be very dramatic in *Appl^d^* but they do have physiological consequences; the loss of APPL resulted in a reduction in the amplitude of evoked excitatory junctional potentials (EJPs) when recording from body wall muscles of larvae (Ashley et al., 2005). Performing whole-cell patch clamp measurements on embryonic cells in culture revealed that both, the loss and overexpression of APPL increased A-type K^+^ currents, suggesting a role of APPL in modulating synaptic function (Li et al., 2004). Additional studies by the same group suggest that this is mediated via the secreted ectodomain (sAPPL) and a similar finding has been made in mammals using cultured hippocampal neurons treated with sAPPα (Furukawa et al., 1996).

A role of APPL in axonal transport was suggested by the finding that overexpression of APPL caused transport defects detectable by the accumulation of vesicles or mitochondria, whereby this phenotype required the presence of the C-terminus (Torroja et al., 1999; Gunawardena and Goldstein, 2001; Shaw and Chang, 2013). Changes in axonal trafficking have also been described after the loss of APPL (Gunawardena and Goldstein, 2001), indicating that the role in axonal transport is a physiological function of APPL. This is also supported by the observation that a dominant-negative mutation of *Drosophila* Tip60, a histone acetyltransferase that has been shown to bind to the C-terminus of APP proteins, also induced axonal trafficking defects (Johnson et al., 2013). In addition, this mutation enhanced transport defects induced by APP. Another manipulation that enhanced the trafficking defects caused by APP and also by APPL is a knock down of *nebula* while overexpression of Nebula suppressed this phenotype (Shaw and Chang, 2013). Manipulating Nebula alone had no effect and therefore its function in axonal trafficking under normal physiological conditions is unclear. Interestingly, Nebula is the fly homolog of Down syndrome critical region 1 (DSCR1) and almost all Down syndrome patients develop AD (Wisniewski et al., 1985). At this point the role of DSCR1 in AD is not understood; however, due to DSCR1 being overexpressed in Down syndrome (Fuentes et al., 2000) one would expect a suppression of possible transport defects caused by the third copy of the APP gene. Interestingly, overexpression as well as loss of Nebula affects synaptic function and memory formation in flies (Chang et al., 2003; Chang and Min, 2009).

## APPL and Neuronal Survival

**Figure 2:**
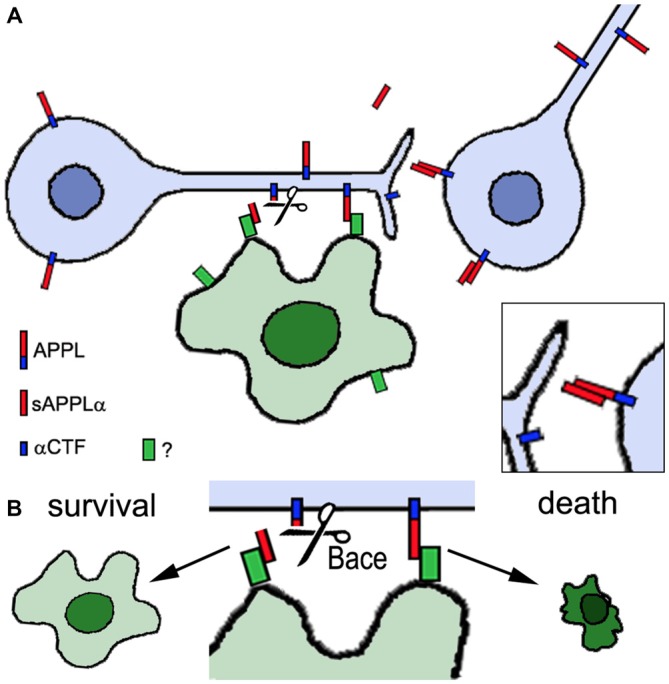
**Proposed role of APPL in neuronal and glial survival.** Neurons express APPL that can be cleaved by α-secretase activity (Kuzbanian, KUZ) resulting in the secretion of sAPPLα (red). sAPPLα binds to full-length APPL, activating an unknown pathway that can promote neuronal survival (inset in **A**). However, full-length APPL can also interact with an unknown glial factor (green). If this interaction is interrupted by *Drosophila* β-secretase (dBACE) cleavage of APPL the glial cell survives whereas increased or ongoing contact between full-length APPL and the glial factor triggers glial death **(B)**. Neurons are shown in blue, the glial cell in green.

The experiments described above reveal that changes in APPL can interfere with neuronal development. But APPL has also been demonstrated to play a role in the integrity of the adult nervous system. *Appl^d^* flies have a significantly reduced life span, shortened to approximately two thirds of the survival span of wild type flies, and they show signs of neurodegeneration when aged (Wentzell et al., 2012). This was detectable by the formation of spongiform lesions in the brains of 3 week old *Appl^d^* flies and although they are not very numerous, such lesions do not occur in age-matched wild type brains. Furthermore, the loss of APPL can aggravate the neurodegeneration caused by mutations in other genes, like *yata* and *löchrig* (*loe)*. Yata belongs to a family of pseudokinases, found in almost all eukaryotes, that play a role in vesicle trafficking of secretory proteins and the export of tRNA from the nucleus (Anamika et al., 2009). *yata* mutant flies show progressive degeneration that affects the brain and retina (Sone et al., 2009). This phenotype was enhanced by the loss of APPL whereas overexpression of APPL ameliorated it, suggesting a neuroprotective function of APPL. Similarly, combining the *loe* mutation with *Appl^d^* significantly worsened the neurodegeneration that is observed in the brain of *loe* mutants (Tschäpe et al., 2002). *loe* encodes the γ-subunit of AMP-activated protein kinase (AMPK), a key enzyme in regulating energy homeostasis (Kemp et al., 1999). AMPK also regulates protein prenylation and *loe* mutant flies show an increase in Rho prenylation and activity and changes in actin dynamics (Cook et al., 2012, 2014). Interestingly, the Rho pathway has also been connected with modulating Aβ production in vertebrates (Tang and Liou, 2007). In contrast to the enhancing effect of the *Appl^d^* mutant, overexpressing APPL suppressed the degeneration in *loe* mutant flies and the same effect was achieved by expressing the secreted sAPPL (Wentzell et al., 2012). However, the latter was only protective in the presence of endogenous APPL and co-immunoprecipitation experiments showed that sAPPL can bind to full-length APPL. This suggests that sAPPL acts as a ligand that binds to full-length APPL as a receptor (Figure 2). The protective function appears to be mediated specifically by the α-cleaved ectodomain because additional expression of Kuzbanian (KUZ) was also protective (Wentzell et al., 2012). KUZ is homologous to ADAM10 and like its vertebrate ortholog it acts as an α-secretase (Carmine-Simmen et al., 2009). In contrast, increasing β-cleavage by inducing *Drosophila* β-secretase (dBACE; Bolkan et al., 2012) expression enhanced the degeneration in *loe*. A neuroprotective function of the α-cleaved sAPP was also described in mice and like in flies it required the presence of full-length APP (Milosch et al., 2014). Together with findings that expression of APPL ameliorated the degenerative phenotype in a *Drososophila* RasGAP (*vap*) mutant and flies mutant for the microtubule binding protein MAP1B (*futsch^olk^*) (Wentzell et al., 2012), this further supports a neuroprotective function of APP proteins and their α-cleaved ectodomains. Interestingly, in the case of *loe* a reduction in sAPPLα may be part of the mechanism leading to the degenerative phenotype in this mutant because *loe* mutant flies showed a decrease in APPL processing whereas additional LOE expression promoted cleavage (Tschäpe et al., 2002).

That the cleavage and generation of specific fragments is important for the protective function is also supported by studying mutations in proteins that affect APPL processing. Transmembrane and Coiled-coil domain 2 (TMCC2) is a vertebrate protein that can form a complex with APP and ApoE and promote APP cleavage (Hopkins et al., 2011). Its *Drosophila* homolog is encoded by *dementin (dmtn)* and loss of neuronal DMTN caused neuronal degeneration in the adult brain and a reduced live span (Hopkins, 2013). It also interfered with the processing of APPL, resulting in the production of an abnormal 50 kD fragment. Similarly, the loss of dBace in photoreceptors resulted in degeneration but in this case of glial cells in the lamina, the main target region of photoreceptors (Bolkan et al., 2012). That this is indeed due to an effect on APPL and not another target of dBace was shown by the result that this phenotype was suppressed in the *Appl^d^* background. In contrast, expressing secretion-deficient APPL (APPL^sd^) enhanced the glial degeneration, supporting the hypothesis that cleavage of APPL is required for glial survival. These findings reveal that APPL not only plays a role in the survival of both, neurons and glia. However, for glia additional full-length APPL seems to be deleterious and the cleavage by dBACE prevents the glial cell death (Figure 2).

Lastly, APPL was found to be upregulated after injury (Leyssen et al., 2005). However whether this is connected to a protective mechanism, like a possible axonal sprouting of neighboring neurons after neuronal loss, is so far unclear. Although an upregulation of APP after injury has also been observed in mammals, this mostly seems to have negative consequences because it can increase the risk to develop AD or other neurodegenerative diseases (Shi et al., 2000; Gupta and Sen, 2016; Ułamek-Kozioł et al., 2016).

## Behavioral Deficits and APPL

As mentioned before, changes in APPL levels also affect behavior, including memory (see accompanying review by V. Goguel). Furthermore, *Appl^d^* flies also show a significantly reduced performance in the fast-phototaxis assay (Luo et al., 1992), a test that can be used to measure general fitness, locomotion, and visual orientation (Benzer, 1967). The phototaxis phenotype may be due to the loss of secreted APPL fragments because expression of full-length APPL could restore this function whereas secretion-deficient APPL^sd^ could not (Luo et al., 1992). Interestingly, also the overexpression of APPL induced phototaxis phenotypes that were further enhanced by expression of dBACE (Carmine-Simmen et al., 2009). The latter suggests that the deficits in the phototaxis assay after APPL overexpression are due to the generation of the neurotoxic dAβ cleaved from the full-length protein. This is supported by the finding that expression of only dAβ also causes phototaxis defects that are even more severe (Carmine-Simmen et al., 2009). In the case of APPL overexpression, the behavioral deficits could be a consequence of the degeneration and neuronal cell death that is detectable after APPL expression. In contrast, the *Appl^d^* deletion mutant shows very subtle morphological changes and modestly increased cell death is only detectable late in life. Therefore the loss of APPL may directly interfere with neuronal function, possibly by affecting synaptic functions.

Finally, recent experiments suggest a function of APPL in the regulation of circadian rhythms, due to the observation that increasing APPL levels prevented the age-related decline in rhythmicity (Blake et al., 2015). This function seems to be specifically mediated by the full-length protein because expressing additional dBACE or KUZ resulted in a disruption of the rhythmic activity pattern. In addition to supporting a protective role for the full-length APPL this also indicates that a cleavage product is deleterious for rhythmicity. Because dBACE and KUZ expression disrupted the circadian activity pattern, this appears to be due to a fragment produced by both cleavage events, excluding dAβ and the N-terminal fragment. However, both β- and α-cleavage promote processing by the γ-secretase and therefore the production of the AICD. Confirming the role of the AICD in circadian rhythmicity, expressing only the AICD pan-neuronally or specifically in the central pacemaker neurons disrupted rhythmicity in an age-dependent manner (Blake et al., 2015). Like humans, flies are diurnal animals and this rhythmicity is regulated by the circadian clock. The clock generates a *circa* 24 h periodicity by an autoregulatory negative feedback loop of four core clock genes and their proteins; Clock and Cycle (BMAL1 in mammals) are the positive elements which promote transcription of the negative elements Period and Timeless (Hardin and Panda, 2013). These proteins are transcriptional regulators that generate circadian rhythms in downstream clock-controlled genes, providing a temporal coordination of cellular and physiological processes with the environment. Supporting a direct role of APPL in regulating circadian rhythms, altering the cleavage pattern of APPL interfered with the rhythmic expression pattern of Period in the central pacemaker cells while not affecting the survival of these neurons (Blake et al., 2015). Because the AICD has been shown to play a role in transcriptional regulation in vertebrates (Cao and Sudhof, 2004; von Rotz et al., 2004), this function of APPL may be an effect of the AICD on the transcription of Period. Not being a transcription factor itself, the AICD forms a ternary complex Fe65 and Tip60. Intriguingly, the loss of *Drosophila* Tip60 induces sleep disturbances and reduces the axon length of central pacemaker neurons (Pirooznia et al., 2012), providing another hint that the AICD may regulate the circadian clock and rhythmicity.

## Conclusion

The studies described above show that full-length APPL can act as a receptor that promotes neurite growth and synaptogenesis *in vivo*. This function appears to require the C-terminus which, together with various interaction factors, can activate downstream signaling pathways, similary to what has been suggested for vertebrate APP (Deyts et al., 2016). For some of these neurodevelopmental functions, cell adhesion molecules like Fas II may act as the activating signals. Fas II has been shown to be required for the function of APPL at the larval NMJ but Fas II is also enriched in mushroom body neurons. Therefore, an interaction between APPL and Fas II might also be required for the correct formation of the mushroom body lobes. Because the mushroom body neurons are crucial for memory formation, this raises the possibility that the Fas II-APPL interactions take part in synaptic plasticity and memory formation, an issue that has not been explored so far.

However, APPL can also act as a ligand via its secreted ectodomains, whereby the α- vs. the β-cleaved fragment seem to play different, even opposing roles. Expression of the secreted sAPPL promotes correct α-lobe formation in *Appl^d^* mutants and neuronal survival in *loe*, whereby the protective function appears to be mediated specifically by the α-cleaved ectodomain whereas the β-cleaved form is neurotoxic. Such opposing functions of the ectodomains have also been described in vertebrates with sAPPα connected to neuroprotective functions (Araki et al., 1991; Mattson et al., 1993; Goodman and Mattson, 1994) whereas sAPPβ was shown to be deleterious for neuronal survival (Nakagawa et al., 2006; Nikolaev et al., 2009). Lastly, the experiments in *Drosophila* showed that APPL can activate its receptor function by binding to its own ectodomain and recently, a similar finding was reported for mammals where sAPPα protected cells from serum-starvation induced cell death only in the presence of full-length APP (Milosch et al., 2014).

Although the studies in *Drosophila* and other models have provided important insights into the functions of APP proteins and their fragment, we are still far away from understanding the various roles of this protein. *Drosophila* provides a variety of tools and assays to study the physiological functions of APP proteins *in vivo* and future experiments including these model will hopefully unravel the functions of APP and the pathways it is involved in. In turn, this can then provide the basis to determine whether and how disruptions of these functions contribute to the deleterious effects seen in Alzheimer patients.

## Author Contributions

MC provided literature. DK wrote review.

## Funding

This work was supported by the National Institute of Health, NINDS project grant (NS096332).

## Conflict of Interest Statement

The authors declare that the research was conducted in the absence of any commercial or financial relationships that could be construed as a potential conflict of interest.
